# The numerical classification and grading standards of daylily (*Hemerocallis*) flower color

**DOI:** 10.1371/journal.pone.0216460

**Published:** 2019-06-06

**Authors:** Huliang Cui, Yanan Zhang, Xiaolu Shi, Feifei Gong, Xiong Xiong, Xiuping Kang, Guoming Xing, Sen Li

**Affiliations:** 1 College of Horticulture, Shanxi Agricultural University, Taigu, China; 2 Facility Horticulture Enginering and Technology Center of Shanxi Province, Taigu, China; United States Department of Agriculture, UNITED STATES

## Abstract

This study collected 183 *Hemerocallis* varieties to conduct numerical classification of flower color and provide valuable baseline data and foundational theory for normalization and precision of *Hemerocallis*. The color CIELab phenotypes were collected via colorimeter (CR-10 Plus), which separately measured three sepal and petal parts (throat, eye and limb). The colors of experimental samples were artificially named by the Royal Horticultural Society Colour Chart (RHSCC). All the data were analyzed using R software. The results showed that the throat was predominantly green-yellow, light yellow and yellow; green-yellow accounted for the largest proportion of sepals (67.76%) and petals (69.40%). The eye was more abundant, and there were significant differences between sepals and petals. The limb was clustered into five color groups (orange, yellow, pink, red and purple); the yellow group had the most varieties for both sepals and petals, containing 57.38% and 55.74%, respectively. Both sepals and petals had significant differences (*p*<0.0001) in color (△E), redness (a*) and color angle (*h*) for the throat, eye and limb. However, the difference in CIELab phenotypes between the eye and limb were not significant. According to “Dual Classification”, the color classification standard was proposed as a 3-level standard. The color of sepal and petal consistency served as the first standard, and the color of limb was the second standard. The color pattern types of pure, gradual change, watermark and eye spot, served as the third standard. It has been proposed that all the 183 experimental varieties were divided into two categories, five groups and finally four types. This study provides a classification basis and reference for numeric and standardized color phenotype description for *Hemerocallis*.

## Introduction

The Daylily (*Hemerocallis* spp.) is one of the most famous ornamental crops in the world. The genus *Hemerocallis* consists of 14 wild species; 11 of them originated from China [1, 2]. Over 83,000 modern cultivars [3] are widely planted for ornamental, vegetable or medicinal use. *Hemerocallis* has application value, divided into edible day lily varieties and horticultural varieties. In Chinese, the edible day lily is known as “Jin zhen” (golden noodle), which was a traditional ingredient in soup and stir fry [4]. The complex genetic background of *Hemerocallis* requires quantitative classification of flower color phenotypes to promote the standardization of description in germplasm resources and efficient utilization of resources.

The classification of *Hemerocallis* varieties were first reported by A.B. Stout [5], who identified 15 color patterns for the floral organs. Since then, more researchers have focused on variety classification. Xiong et al. [6] placed the 11 *Hemerocallis* taxa into 4 clusters using cluster analysis and principal component analysis. For example, *H*. *lilioasphodelus*, *H*. *citrine*, *H*. *thunbergii* and *H*. *multiflora* were grouped in the first cluster; *H*. *dumortieri* and *H*. *middendorfii* in the second cluster; *H*. *plicata*, *H*. *nana* and *H*. *forrestii* in the third cluster; and other cultivars in the fourth cluster. Kong [7] found that seed micromorphology of *Hemerocallis* could be used in germplasm classification at the species level; however, Li et al. [8] found the ploidy analysis difficult for the complex ploidy of *Hemerocallis* cultivars, when the ploidy of many common cultivars was unknown. Saito et al. [9] studied the ploidy of 9 wild species and 94 cultivated varieties by flow cytometry methods; 59 diploid varieties, 2 triploid varieties and 33 tetraploid varieties were finally identified. Saito et al. [9] also reported that: *H*. *lilioasphodelus*, *H*. *thunbergii* and two varieties of *H*. *dumortieri* were diploid; *H*. *fulva* var. *kwanso* was triploid; and *H*. *fulva* var. *littorea*, *H*. *fulva* var. *longituba* and *H*. *fulva* var. *rosea* were diploid. Thus, *H*. *fulva* was a species with a diverse genetic background.

This complicated genetic background made it difficult to classify *Hemerocallis*. Thus, many researchers categorized daylily germplasm differently. Xiong et al. [10] divided *Hemerocallis* into a day-blooming group and a night-blooming group. Chinese scholars always use “dual classification” to classify flower varieties [11], where both evolutionary and development tendency as well as practical application and morphological characteristics are considered. Du et al. [12] proposed five classification criteria for *H*. *hybridus* according to its breeding strategy, i.e., gene type, plant type, length of green period, early or late flowering period and flower characteristics. However, this research did not characterize the flower color of *H*. *hybridus* in detail. Zhu et al. [13] developed 8 grading standards by investigating 273 varieties. In this research, stable hereditary traits such as chromosome number and flowering habit were used as the first and second grading standards; flower color was used as the fifth grading standard, without consideration of flower color numerical treatment nor color pattern. Wild day lily germplasm always showed a single flower color, whereas modern hybrid horticultural varieties always showed a more complex color distribution pattern. Wang et al. [14] separately classified pure, mixed, poly-color, multi-color and double-color for floral organ color, and they also identified color spot, watermark, middle rib and throat color patterns. However, the main ornamental part of daylily floral organs was the six perianth lobes, which are commonly called the outer three perianth lobes and the inner three perianth lobes. The outer three perianth lobes belong to sepals and the inner three perianth lobes belong to petals [15]. It should be noted that the color names (e.g., color spot, watermark and throat) indicated perianth lobe color not floral positions, which were also easily confused in previous studies. In addition, the difference between outer and inner perianth lobes for daylily floral organs had not been reported.

Flower color is an important phenotypic trait for classification [16] of ornamental plant varieties. Although flower color can be determined using colorimetric cards, subjective error can limit the industry interchange. Thus, measuring color phenotypes using instrumental color measurement has been used for many ornamental plants, such as chrysanthemum [17], rose [18] and carnation [19]. To date, there are no studies that report quantitative flower color for *Hemerocallis*.

In this study, we collected 183 *Hemerocallis* varieties from 2016 to 2018 and focused on quantitative analysis of flower color using a colorimeter (CR-10 Plus). The outer and inner perianth lobes were separated and measured for color phenotypic data, respectively. The perianth lobes contained three different parts (throat, eye and limb), the colors of which were named using the Royal Horticultural Society Color Chart (RHSCC). We subsequently proposed flower color classification standards for *Hemerocallis* according to “Dual Classification” [11]. This study also attempted to provide a basis for the precise definition of flower color and laid a foundation for *Hemerocallis* flower color breeding.

## Materials and methods

### Materials

We collected 183 daylily varieties cultivated in Taigu County of Shanxi province (E: 112.53°, N: 37.42°) and investigated flower color data continuously from 2016 to 2018. These varieties contained 14 wild species, 12 breeding lines from our hybrids, 63 edible day lily landraces and 58 horticultural cultivars from China, and 36 Euro-American cultivars from the US, Canada, Netherlands, and Austrians (S1 Table).

### Flower color measuring method

**Fig 1:**
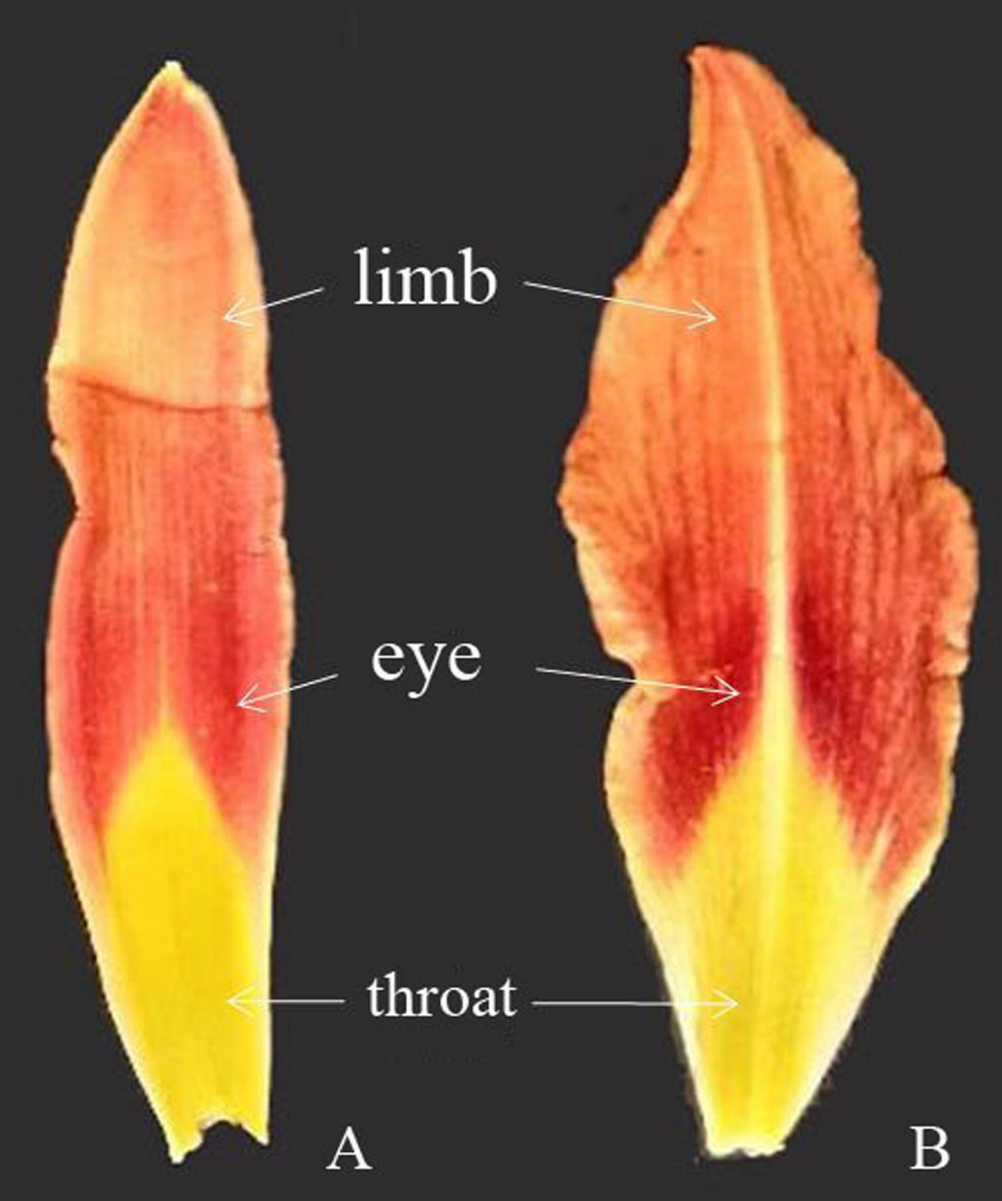
The illustration of daylily flower color measurement location (A: sepal, B: petal).

In this study, five blooming flowers were randomly selected from each variety. The outer and inner perianth lobes of each flower were separately placed on clean white paper for color data measurement using a colorimeter (CR-10 Plus). As shown in Fig 1, the three parts of the outer and inner perianth lobes (throat, eye and limb) were measured under the following conditions: built-in light source D65°; window diameter 8 mm; and observation angle 10°. Color indicators, such as lightness (L*), redness (a*), yellowness (b*), total aberrations (△E), chroma (*C*) and hue angle (*h*), were measured. The average of each color indicator was used to represent the color information for each part. In our experimental operation, the position of the middle rib was avoided whenever possible to minimize the interference of the color difference between the middle rib and other parts.

### Color distinction and data analysis

We artificially distinguished and named the color of different parts according to RHSCC, and recorded petal color pattern by visual inspection. The pattern of pure was labeled as 1, gradual change was labeled as 2, watermark was labeled as 3, eye spot was labeled as 4. The middle rib was labeled as 0 or 1. The experimental data measured by colorimeter was exported to Microsoft Excel 2007. All statistical analysis and graphics works were calculated using R Language, such as cluster analysis, nonparametric test and statistical figures.

## Results

### The cluster analysis of color phenotype

The cluster analysis for the color phenotypic data from three parts of outer and inner perianth lobes (throat, eye and limb) were analyzed using R Language. First, the values of L*, a* and b* were standardized by Euclidean methods under the dist() function. Second, the standardized data was calculated under the hclust() function using the parameter “method = complete”. After drawing jump lines at H = 40, the cluster results of the three parts from sepals and petals were corrected according to RHSCC. The cluster results are shown in Table 1.

**Table 1:** The clusters of *Hemerocallis* sepal and petal color phenotypes.

Measured Part	Color Group	No. of Species		Percentage(%)		RHSCC Color Name
		Sepal	Petal	Sepal	Petal	
Throat	yellow-green	104	127	67.76	69.40	150A~150C、154A~154D
	light yellow	77	48	27.87	26.78	2B、3B、4B
	yellow	2	8	1.09	4.37	4A、5A~5B、6A~6C
Eye	yellow-green	38	—	20.77	—	150A~150C、154A~154D
	light yellow	105	86	57.38	46.99	2B、3B、4B
	yellow	6	40	3.27	21.87	4A、5A~5B、6A~6C
	light pink\light red	19	—	10.38	—	56A~56D、65A~65D、69A~69D
	orange	—	13	—	7.10	17A~17C、23A~23C、25A、N25A~N25D、28A~28B、30A~30D
	red \light purple	15	22	8.20	12.02	N30A、40A~41D、N57D、58D
	dark red\purple	—	22	—	12.02	45A~47B、59D、83A~83C
Limb	yellow	105	102	57.38	55.74	2B~6C、150A~150C、154A~154D
	pink	12	18	6.56	9.84	65A~65D、69A~69D
	orange	30	24	16.39	13.11	17A~17C、23A~23C、25A、N25A~N25D、28A~28B、30A~30D
	red	23	26	12.57	14.21	N30A、40A~41D、45A~47B、N57D、58D
	purple	13	13	7.10	7.10	59D、83A~83C

#### The cluster analysis of color phenotype in the three parts of sepals

There were three colors distributed in the throat samples in this experiment: yellow-green, light yellow and yellow. The largest proportion of samples was colored yellow-green (67.76%), followed by light yellow (27.87%), and the smallest proportion of samples was colored yellow (1.09%).

The color of the eye was significantly richer than the throat. There were five cluster groups (Table 1) such as yellow-green, light yellow, yellow, light pink/light red and red/light purple. Most samples were light yellow (57.38%), whereas yellow accounted for the lowest proportion (3.27%).

It must be noted that the limb colors were clustered into six groups at H = 40 after cluster analysis. However, we found that the light yellow group was most similar to the yellow group according to RHSCC. In addition, both edible day lily and horticultural cultivars were observed to have these two color groups at the same time. Results showed that there were 5 cluster groups, such as orange, yellow, pink, red and purple (Table 1), after merging light yellow and yellow into the yellow group.

#### The cluster analysis of color phenotype in the three parts of petals

Similar to sepals, the colors of the throat petals were yellow-green, light yellow and yellow, with proportions of 69.40%, 27.87% and 4.37%, respectively. However, the colors of petal eyes were inconsistent with those of the sepals, which are shown in Table 1. When compared with sepals, the two petal color groups of yellow-green and light pink/light red were lacking, whereas the two groups of orange and dark red/purple were added (Table 1). The largest proportion observed was light yellow (46.99%) and the smallest proportion was orange (7.10%). Hence, the color of eye variation of *Hemerocallis* was more complex than that of the throat because the eye belongs to the transitional position between throat and limb.

Petals were the main ornamental part of daylily flower organs. The cluster results showed five color groups (orange, yellow, pink, red and purple) which were similar to sepals. The 183 germplasm samples were divided into the five color groups in this study. The largest proportion of samples belonged to the yellow group (55.74%), followed by orange group (13.11%), and the smallest proportion belonged to the purple group (7.10%).

### The difference in color phenotype among different parts

The color phenotypic values for different parts were different. The data between sepals and petals were calculated by the Q-Q normality test on the R Language platform, but only C value for sepal throat and the △E value for sepals and petals showed a normal distribution (S1, S2, S3, S4, S5 and S6 Figs). Hence, this study used the Mann-Whitney U nonparametric test for further analysis (Table 2).

**Table 2:** The *p* value of the Mann-Whitney U test between different parts of sepal and petal color.

Contrast Group		CIELab Coordinate					
		ΔE	L*	a*	b*	*C*	*h*
Sepal	Ⅰ	4.87e-09***	0.045*	8.76e-14***	0.083	3.04e-05***	4.476e-05***
	Ⅱ	1.69e-07***	0.109	5.45e-14***	0.818	0.074	1.379e-06***
	Ⅲ	0.568	0.119	0.889	0.040*	0.037*	0.217
Petal	Ⅰ	2.36e-11***	0.394	1.314e-11***	0.306	0.010*	0.000225***
	Ⅱ	1.85e-12***	0.275	4.742e-14***	0.073	0.0001244***	2.47e-05***
	Ⅲ	0.254	0.358	0.359	0.764	0.202	0.367
Ⅳ		0.000354***	0.012*	0.129	5.58e-05***	2.12e-05***	0.411
Ⅴ		0.000491***	0.029*	0.380	0.043*	0.025*	0.729
Ⅵ		6.86e-05***	0.0032***	0.108	0.00041***	1.79e-06***	0.941

Note:

*** indicates p<0.001,

** indicates p<0.01,

* indicates p<0.05;

Ⅰindicates throat—eye contrast group, Ⅱ indicates throat—limb contrast group, Ⅲ indicates eye—limb contrast group, Ⅳ indicates sepal throat—petal throat contrast group, Ⅴ indicates sepal eye—petal eye contrast group, Ⅵ indicates sepal limb—petal limb contrast group

#### The color phenotype difference between different parts of sepals

As shown in Table 2, the values for color indicators were different between different parts. Throat showed significantly different ΔE value (at *p*<0.001 level) than the other two parts, whereas there was no difference between eye and limb (*p*>0.05). Similarly, the value of a* was significantly different between throat and other parts (*p*<0.001). The value of L* was significantly different between throat and eye (*p*<0.05). The *C* value was significantly different (*p*<0.001) between throat and eye. But the value of b* showed no difference. Hence, the throat was different from the other parts, whereas the eye and the limb had no significant difference.

### The color phenotype difference between different parts of petals

Contrary to sepals, the three parts of petals showed no difference in flower color phenotype. There were no difference among all three parts for L* and b* (*p*>0.05). The color indicators between eye and limb were also not significantly different (*p*>0.05). Throat was significantly different (*p*<0.001) from the other two parts for values of ΔE, a* and *h*. For the value of *C*, throat was significantly different with eye (*p*<0.05) and limb (*p*<0.001). It indicated significant differences between throat and other parts, but there was no significant difference between eye and limb.

#### The difference in color phenotype among experimental germplasm

**Fig 2:**
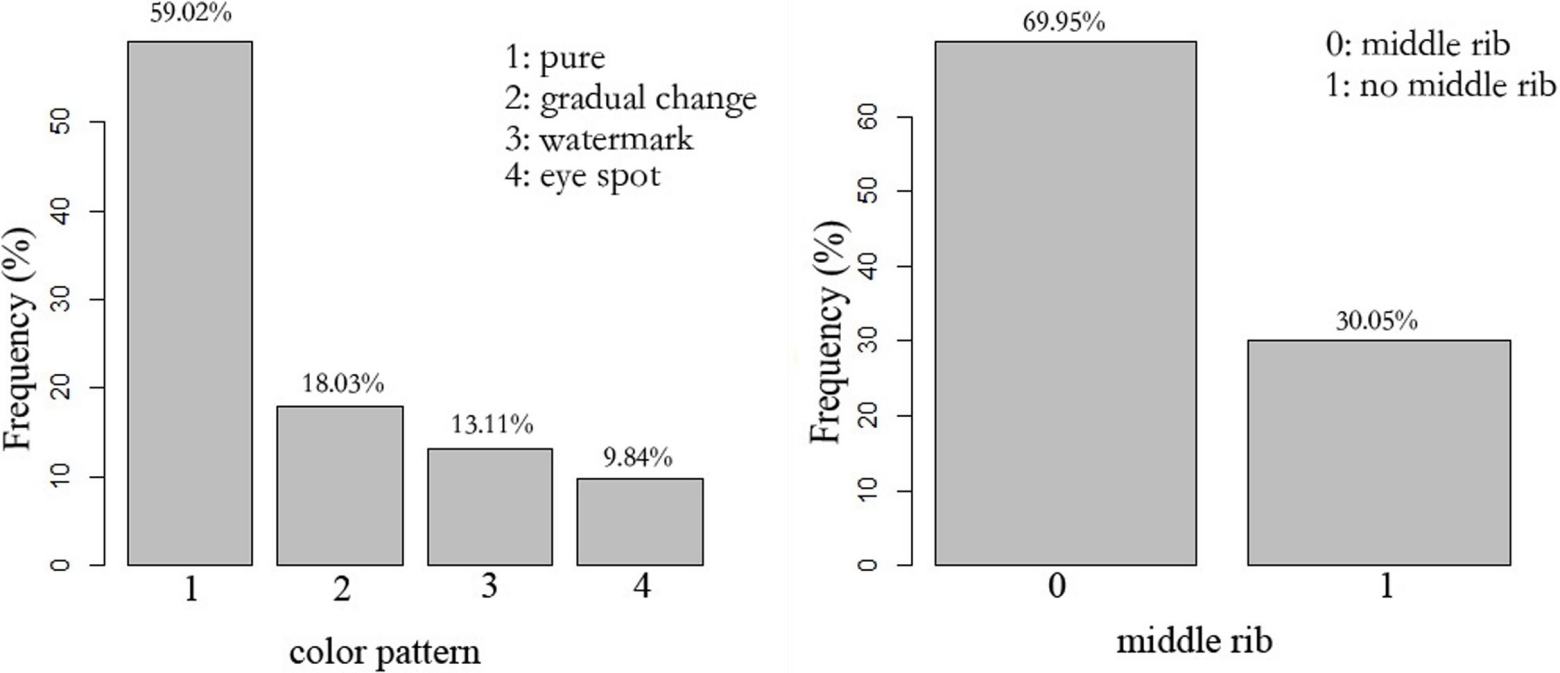
The distribution ratio of color pattern of Hemerocallis.

The germplasm of *Hemerocallis* always shows pure, gradual change, watermark and eye spot in different color patterns, and it shows whether a middle rib is present [14]. In this research, we labeled pure as 1, gradual change as 2, watermark as 3 and eye spot as 4. To record whether a middle rib was present, we labeled 0 as middle rib and 1 as no middle rib. Results indicate that all four color patterns were observed in our investigation. There were 108 varieties of pure color, 33 gradual change, 24 watermark, and 18 eye spot, which accounted for 59.02%, 18.03%, 13.11% and 9.84%, respectively (Fig 2). In addition, there were 55 varieties that had an obvious middle rib and 128 varieties without middle rib, which accounted for 30.05% and 69.95%, respectively.

#### Discrimination and classification of color patterns for Hemerocallis

**Fig 3:**
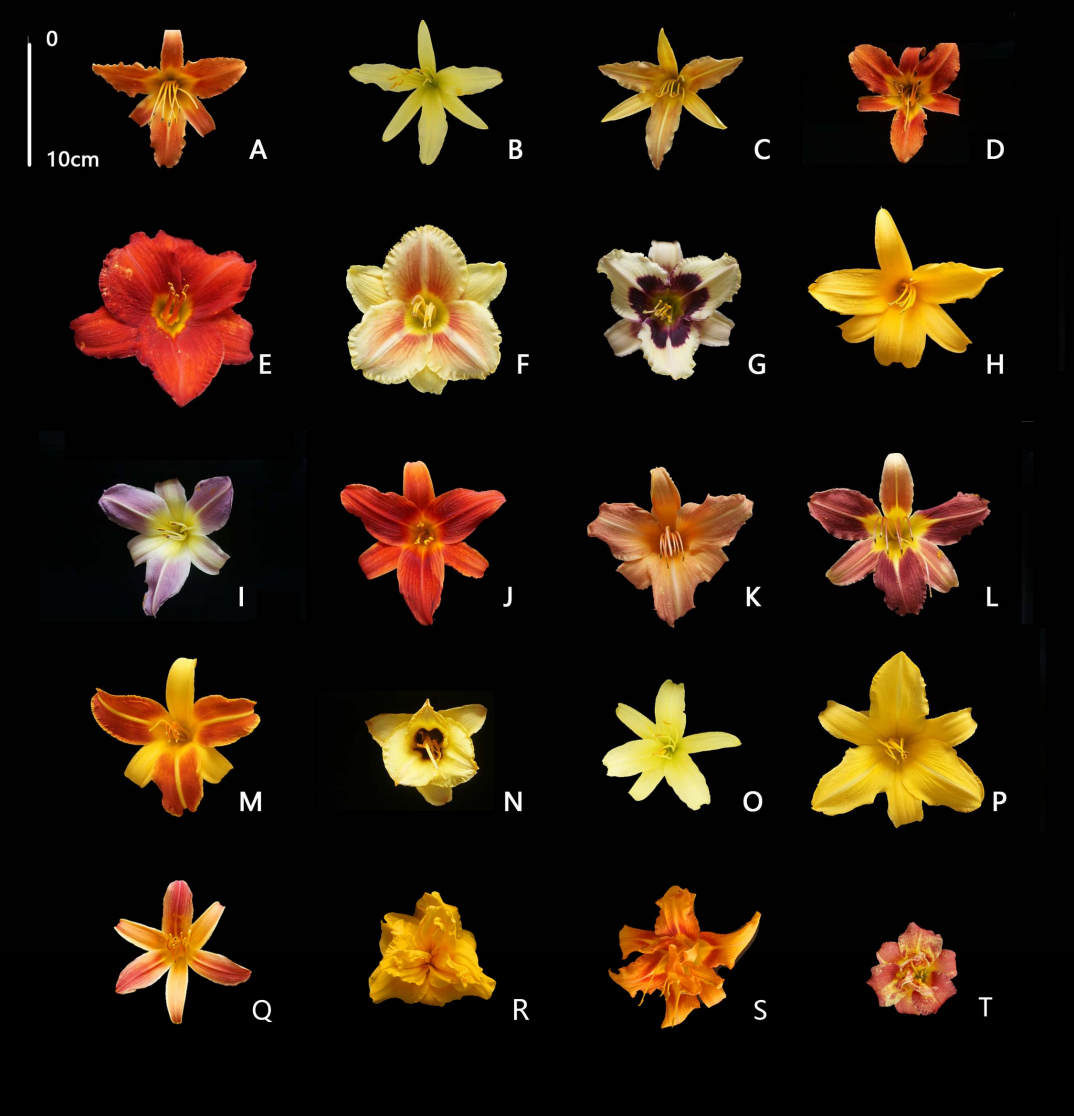
The phenotypic diversity of daylily cultivars flower colors. Note: The cultivars photographed under indoor illumination, are identified as follows: A: ‘Suqian 1-H’, B: ‘ChaZi Hua’, C: ‘Panlong Hua’, D: ‘Suqian 3-C’, E: ‘Apache’, F: ‘Truth’, G: ‘Moon Masquerade’, H: ‘Nakai’, I: ‘Blue Sheen’, J: ‘XiaoHong’, K: ‘Children’s Festival’, L: ‘Elegant Greeting’, M: ‘Frans Hals’, N: ‘Little Bee’, O: ‘Da Wuzui’, P: ‘Ruffled Apricot’, Q:‘Y-326’, R:‘Cream Roll’, S:‘Beijing 1’, T:‘Little Red Baron’.

*Hemerocallis* had abundant flower color phenotypes and the typical samples are shown in Fig 3. The different parts of flower organs could be named according to RHSCC, which are shown in Table 1. In this research, color pattern had been manually observed as pure (Fig 3B, 3H, 3O and 3P), gradual change (Fig 3A, 3D, 3E, 3I, 3K and 3Q), watermark (Fig 3F, 3J and 3K), and eye spot (Fig 3G, 3L and 3N). However, the CIELab data showed no statistical significant difference, particularly for eye spot cultivars. For example, ‘Moon Masquerade’ (Fig 3G) has a significant purple eye spot. The color could be observed to be different between limb and eye, but the Mann- Whitney U results showed no significant difference except the value of L*. In this work, the paired *t*-test (Table 2) also indicated no difference between different parts according to CIELab data. Hence, it was necessary to differentiate between color pattern simultaneously using colorimeter and manual methods. In addition, there were five varieties that had different colors of sepals and petals (Fig 3C and 3M), and they clustered in different groups (Fig 2).

#### Determination of flower color phenotype grading index

Classification of flower varieties always used “Dual Classification” as the reference method [11], which raised “provenances relationship” as the precondition level index. Thus, the traits with evolutionary significance and steady hereditary should be considered preferentially when *Hemerocallis* varieties are classified.

The sepals belong to the calyx, which indicates a different phylogenic relationship when compared with petals belonging to the corolla [15]. In this research, the color indicators were different (*p*<0.05) between sepals and petals at values of ΔE, L*, b* and *C* according to the Mann-Whitney U test results (Table 2). The five varieties, with different colors of sepals and petals, belonged to the bicolor cultivar, which is a peculiar phenomenon among the *Hemerocallis*. Thus, the presence of a color difference between sepals and petals should be viewed as the first classification standard. Therefore, the 183 germplasm could be divided into two categories: bicolor and self-color.

We chose limb color, the main ornamental part of daylily, as the second classification standard, because of the single color of the throat and the lack of difference between the eye and the limb. The 183 germplasms could be divided into five color groups (Table 1). The color pattern discrimination served as the third classification standard, and 4 types could be classified in our study. Finally, the experimental varieties were divided into 2 categories, 5 groups and 4 types, with the following identification.

The Flower color Classification index of *Hemerocallis*

1. Sepal and petal show inconsistency color …………………………………………………… Bicolor category
  2. Limb color is yellow ………………………………………………………………………… Yellow group
    3. Throat, eye and limb colors are identical …………………………………………………… Pure type
    3. Throat color is different from others, but eye and limb are similar …………… Gradual change type
    3. Eye is different from others ………………………………………………………… Watermark type
    3. Eye always has significant spot ………………………………………………………… Eye spot type
  2. Limb color is pink………………………………………………………………………………… Pink group
    3. Throat color is different from others, but eye and limb are similar …………… Gradual change type
    3. Eye is different from others ………………………………………………………… Watermark type
    3. Eye always has significant spot ………………………………………………………… Eye spot type
  2. Limb color is orange…………………………………………………………………………… Orange group
    3. Throat color is different from others, but eye and limb are similar …………… Gradual change type
    3. Eye is different from others ………………………………………………………… Watermark type
    3. Eye always has significant spot ………………………………………………………… Eye spot type
  2. Limb color is red ………………………………………………………………………………… Red group
    3. Throat color is different from others, but eye and limb are similar …………… Gradual change type
    3. Eye is different from others ………………………………………………………… Watermark type
    3. Eye always has significant spot ………………………………………………………… Eye spot type
  2. Limb color is purple………………………………………………………………………………Purple group
    3. Throat color is different from others, but eye and limb are similar …………… Gradual change type
    3. Eye is different from others ………………………………………………………… Watermark type
    3. Eye always has significant spot ………………………………………………………… Eye spot type
1. Sepal and petal show consistent color ………………………………………………………self-color category
  2. Limb color is yellow…………………………………………………………………………… Yellow group
    3. Petal throat, middle and petal color are identical …………………………………………… Pure type
    3. Petal throat was different with others, but middle and petal were same ………… Gradual change type
    3. Middle was different with others……………………………………………………… Watermark type
    3. Middle always has significant spot……………………………………………………… Eyezone type
  2. Limb color is pink………………………………………………………………………………… Pink group
    3. Throat color is different from others, but eye and limb are similar …………… Gradual change type
    3. Eye is different from others ………………………………………………………… Watermark type
    3. Eye always has significant spot ………………………………………………………… Eye spot type
  2. Limb color is orange…………………………………………………………………………… Orange group
    3. Throat color is different from others, but eye and limb are similar …………… Gradual change type
    3. Eye is different from others ………………………………………………………… Watermark type
    3. Eye always has significant spot ………………………………………………………… Eye spot type
  2. Limb color is red……………………………………………………………………………………Red group
    3. Throat color is different from others, but eye and limb are similar …………… Gradual change type
    3. Eye is different from others ………………………………………………………… Watermark type
    3. Eye always has significant spot ………………………………………………………… Eye spot type
  2. Limb color is purple………………………………………………………………………………Purple group
    3. Throat color is different from others, but eye and limb are similar …………… Gradual change type
    3. Eye is different from others ………………………………………………………… Watermark type
    3. Eye always has significant spot ………………………………………………………… Eye spot type

### The characteristic of flower color phenotype

#### The CIELab distribution of different color groups

The color indicators for the limb of petals measured by CR-10 Plus showed significant differences among the five color groups (Table 3). The yellow group had the highest values of L* (45.42~85.10) and b* (29.12~75.78). At the same time, the red group had the lowest value of L* (18.66~60.14), and the purple group had the lowest value of b* (4.30~44.96). The orange group had the highest value of a* (38.50~45.84); meanwhile, yellow and pink groups had the lowest value of a*. The pink group was distributed in 12.48~30.24, and the yellow group had the lowest value (-1.00), which was the only group that contained a negative value among all the color groups.

**Table 3:** The distribution range of CIELab measuring from the limb of petals for *Hemerocallis*.

Color Group	CIELab Coordinate				
	L*	a*	b*	*C*	*h*
yellow	45.42~85.10	-1.00~44.36	29.12~75.78	43.68~79.88	68.10~86.08
pink	61.38~78.58	12.48~30.24	27.66~53.56	30.82~59.74	71.28~78.54
orange	47.34~61.16	38.50~45.84	43.26~58.88	59.34~73.24	67.14~71.90
red	18.66~60.14	14.60~40.30	7.94~47.30	24.00~55.84	63.02~76.42
purple	28.56~59.04	20.32~43.88	4.30~44.96	22.86~60.74	62.98~71.90

**Fig 4:**
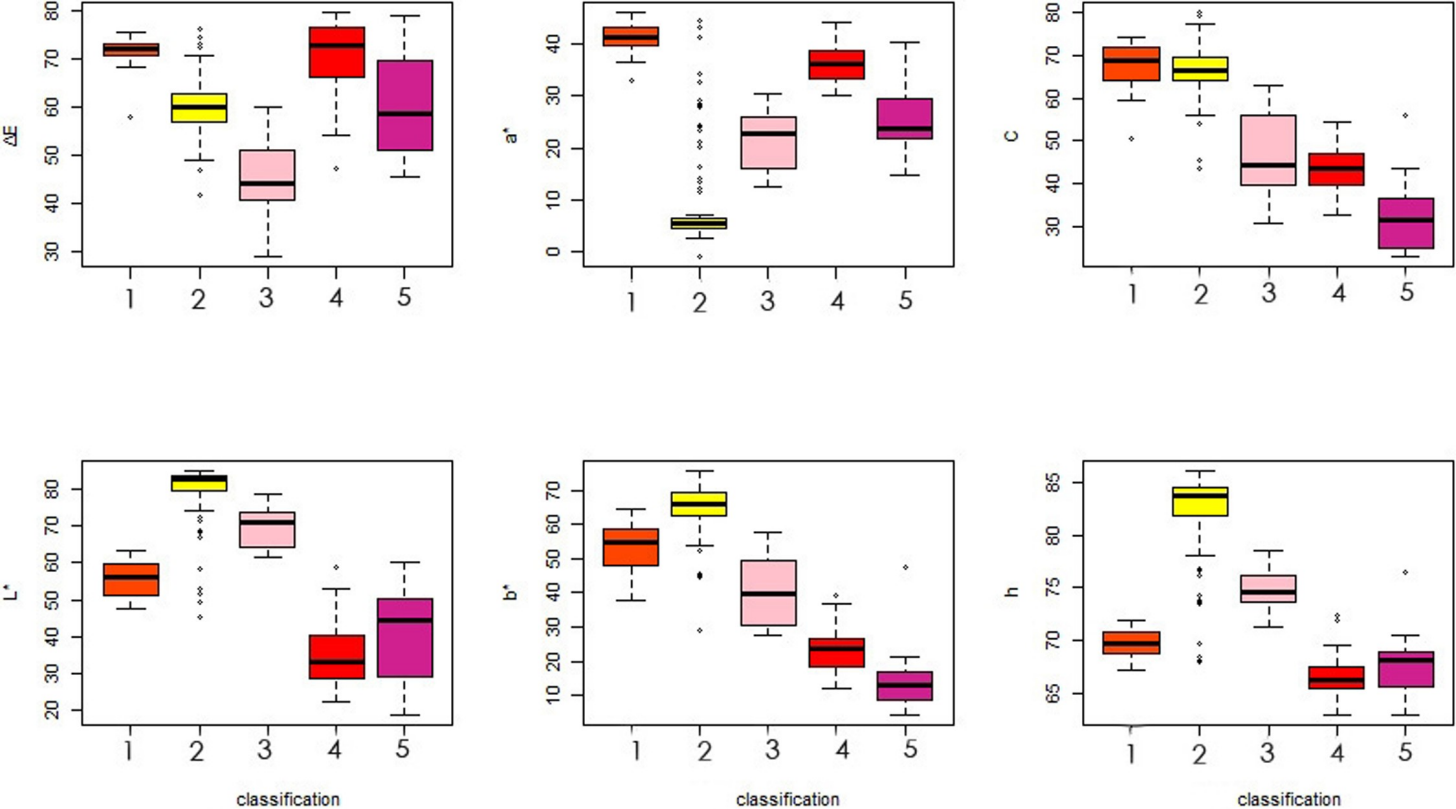
The box plot based on the limb of petal color phenotype for Hemerocallis according to CIELab data. 1: Orange group; 2: Yellow group; 3: Pink group; 4: Red group; 5: Purple group.

The value of ΔE showed significant differences for all five color groups observed in the box plot (Fig 4). Red and purple groups were closed at lower quantile values for L* and *h*. The average of *C* value was similar between red and pink groups, but the pink group was significantly different from the purple group. Red may be the transition color from pink to purple, so these color indicators overlapped. The orange group had many similar characters as the yellow group, indicating common characteristics between the two color groups. After the artificial correction from RHSCC color identification, it could be concluded that the relationship between the color groups and CIELab color phenotype values could objectively distinguish different color groups and basically conform to the characteristics of flower color phenotype for daylily.

#### The color phenotype distribution characteristics of color groups

**Fig 5:**
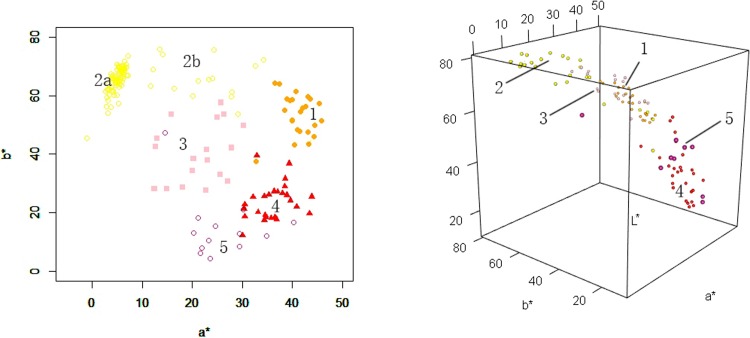
The flower color distribution for limb of petal of Hemerocallis. A: Two dimensional coordinate systems of a*, b*; B: Three dimensional coordinate systems of L*, a*, b*. 1: Orange group; 2: Yellow group (2a mainly contain edible daylily, 2b mainly contain horticultural cultivars); 3: Pink group; 4: Red group; 5: Purple group.

In the two-dimensional coordinate space, where a* rangs from -1.00 to 45.84 on the X-axis and b* rangs from 4.30 to 75.78 on the Y-axis, the experimental varieties were widely distributed (Fig 5). The yellow group was significantly higher and more centered than other groups for the b* value, but it was scattered for the a* value. These results occurred because the yellow group contained two cultivar groups: edible day lily and horticultural cultivars. Edible day lily was mainly distributed in the upper left of the two-dimensional coordinate space (Figs 5A and 2A). Horticultural cultivars were dispersed above the two-dimensional coordinate space (Figs 5A and 2B). Fig 5 shows that the orange group was distributed in the upper right with high values for a* and b*. The purple group mainly distributed below the coordinate axis with lower b* values. There was an outlier represented by ‘Elegant Greeting’ (Fig 3L) that belonged to the purple group according to RHSCC, however the CIELab phenotype was close to the pink group. The results indicated the importance of RHSCC for color discrimination. The red group was distributed overlapping among the orange group, pink group and purple group, which indicated a transitional relationship for red cultivars to orange, pink and purple cultivars of *Hemerocallis*. The three-dimensional coordinate space for L*, a* and b* showed that all 5 groups were distributed throughout the spaces (Fig 5B).

**Fig 6:**
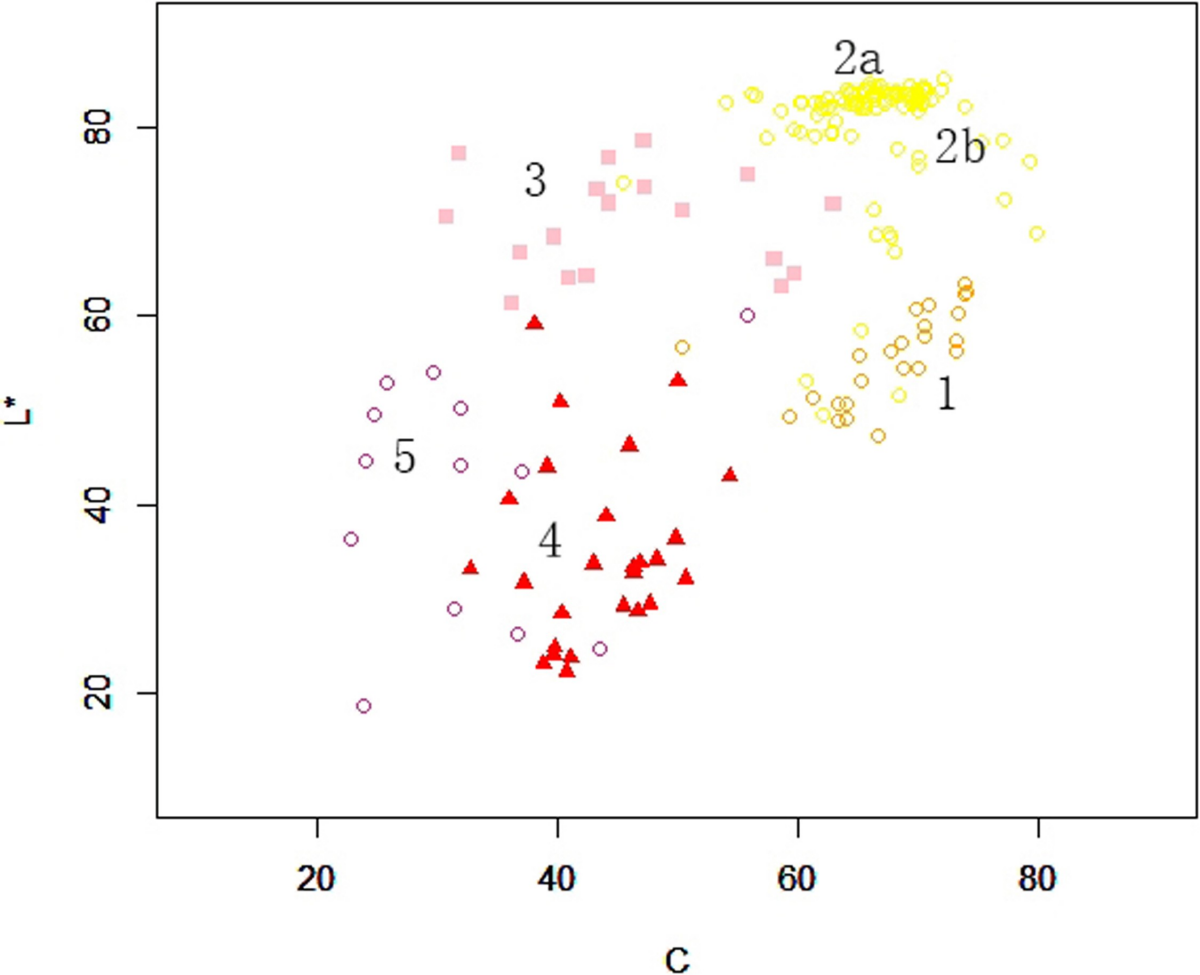
The scatter plot according to L* and C for the limb of petals among Hemerocallis. 1: Orange group; 2: Yellow group (2a mainly contain edible daylily, 2b mainly contain horticultural cultivars); 3: Pink group; 4: Red group; 5: Purple group.

This research also performed regression analysis for L* and *C*; the results are shown in Fig 6. It could be seen that 183 experimental varieties showed a linear relationship; the linear regression equation was y = 28.87+0.45x (R^2^ = 0.4138, *p* = 2.2e-16, *F* = 127.8). It suggest that the L* value increased to a small extent with the increase of the *C* value, but it was not significant. However, the linear regression for the orange and yellow groups were significant; the linear regression equation were y = 27.11 + 0.73x (R^2^ = 0.3823, *p* = 0.0012, *F* = 13.62) and y = 54.24 + 0.15x (R^2^ = 0.044, *p* = 0.0352, *F* = 4.561), respectively. However, the other three color groups (red group, pink group and purple group) had no linear relationships. These results indicated that the values of L* and *C* could not be used for cultivar classification of *Hemerocallis*.

## Discussion

The color phenotype measurement by colorimeter showed deviation and outliers and did not fully characterize the phenotypic characteristic of flower color. Hence, a colorimetric card was needed for revision. A similar phenomena has been described for other ornamental plants such as chrysanthemum [17] and rose [18]. In our study, the experimental samples contained common germplasms of *Hemerocallis*, mainly from Shanxi, Shaanxi, Heibei, Shandong, Hunan, Hubei, Fujian, Sichuan, Yunnan, Gansu, Ningxia, Inner Mongolia and Taiwan. Commercial Euro-American varieties from 4 different countries were also included in this work. We examined 183 daylily varieties by colorimeter in this work, which was the largest number of varieties so far and could provide important information for cultivar classification work for *Hemerocallis*.

In previous studies, Du et al. [12] divided 21 cultivars into 2 categories, 4 series and 8 cultivar groups; the second grading standard “series” means corolla diameter. However, Zhu [13] divided 10 cultivars into 2 categories, 2 series, 2 sects and 5 color groups; the second grading standard “series” indicates florescence, and flower color served as the fourth grading standard. The same traits were examined with different grading standards in different reports, which was not advantageous for cultivar classification and industry communication. In addition, we discovered that the colors of outer and inner perianth lobes showed different genetic expression. The ‘Y-326’ (Fig 3Q) has different colors for sepals and petals; this line represents cross breeding from the female parent ‘Datong’ (yellow) and the male parent ‘Lullaby Baby’ (pink) (cross parents graphic not shown). Thus, the inner and outer bicolor was steady and hereditary. In this study, it was necessary to choose whether the color difference between sepals and petals as the first grading standard. The genetic mechanism for outer and inner perianth lobe colors for *Hemerocallis* needs to be explored in depth in the future, which could benefit breeding of bicolor varieties and enrich the germplasm resources for *Hemerocallis*.

The color pattern of *Hemerocallis* should be determined using traditional methods. No relationship was observed between throat, eye and limb from the CIELab phenotype in this research. In addition, the middle rib cannot be determined by colorimeter because its shape is always a thin line (Fig 3D, 3I and 3L). In this study, the colorimeter window was 8 mm diameter, so the middle rib could not be accurately detected with the window. In addition, we avoided part of the middle rib when measuring flower color to minimize color interference.

In this research, 183 varieties were divided into 5 color groups, without white and blue containing. This may explain why values of L*, a* and b* were distributed narrowly in the two-dimensional coordinate space. Huang [20] reported a* values ranging from -12.00 to 43.56 after measuring white cultivars, which indicates that daylily had a negative value distribution similar to chrysanthemum[17] and rose [18]. *Hemerocallis* has been lacking in blue varieties, but its flower is rich in flavonoids [21], which belong to the phenylpropane metabolic pathway. Anthocyanin also belonged to this metabolic pathway [22]. Blue flower color was monitored by *F3’5’H* delphinidin accumulation [23]. Delphinidin glycoside always expressed purple or dark red alone, but showed a blue color when flavonoids appeared as auxiliary pigments. The main auxiliary pigments were kaempferol and quercetin, which had been observed in *Geranium wilfordii* and *Pelargonium hortorum* [24, 25]. It has been reported that daylily is rich in kaempferol and quercetin [26], which indicates that it has the ability to produce blue cultivars. This work also found many purple and dark red cultivars from the 183 experimental germplasms. If the metabolic mechanism for anthocyanin glycoside and the biosynthesis pathway for delphinidin could be determined, breeders could achieve the objective of breeding blue varieties of *Hemerocallis* in the future. In addition, the single flower of *Hemerocallis* was divided into seven stages, from opening to withering, which usually lasted only 36h [27]. Therefore, its flower organ opening and programmed death was typical [15, 28], which highlights the need for further study. It could be inferred that the daylily was an ideal subject for studying the mechanism of anthocyanin metabolism and molecular regulation in plants.

## Supporting information

S1 Fig The Q-Q normality test of throat of sepal. (TIF)

S2 Fig The Q-Q normality test of eye of sepal. (TIF)

S3 Fig The Q-Q normality test of limb of sepal. (TIF)

S4 Fig The Q-Q normality test of throat of petal. (TIF)

S5 Fig The Q-Q normality test of eye of petal. (TIF)

S6 Fig The Q-Q normality test of limb of petal. (TIF)

S1 Table The experimental *Hemerocallis* germplasms in this study. (DOCX)
