# Supplementary figures and images for: The numerical classification and grading standards of daylily (Hemerocallis) flower color
Source: PLoS One. 2019 Jun 6;14(6):e0216460. doi: 10.1371/journal.pone.0216460 (PMC6553707; doi:10.1371/journal.pone.0216460)

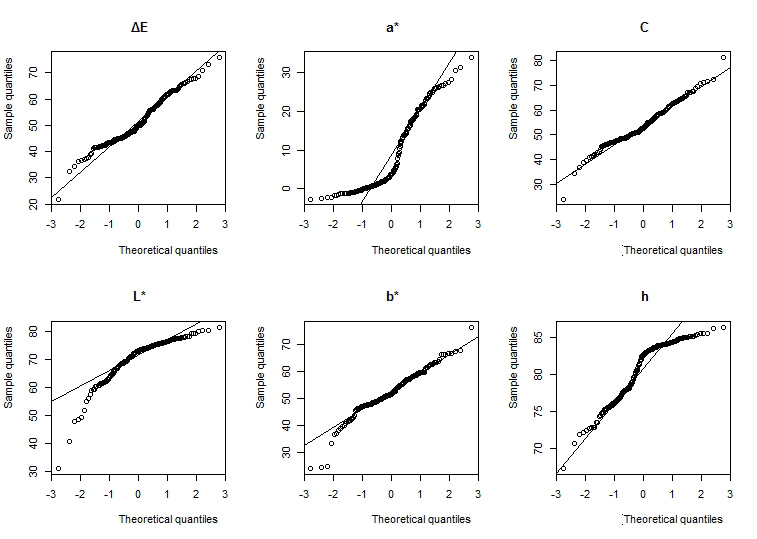

Supplement: S1 Fig — (TIF) [file pone.0216460.s001.tif]

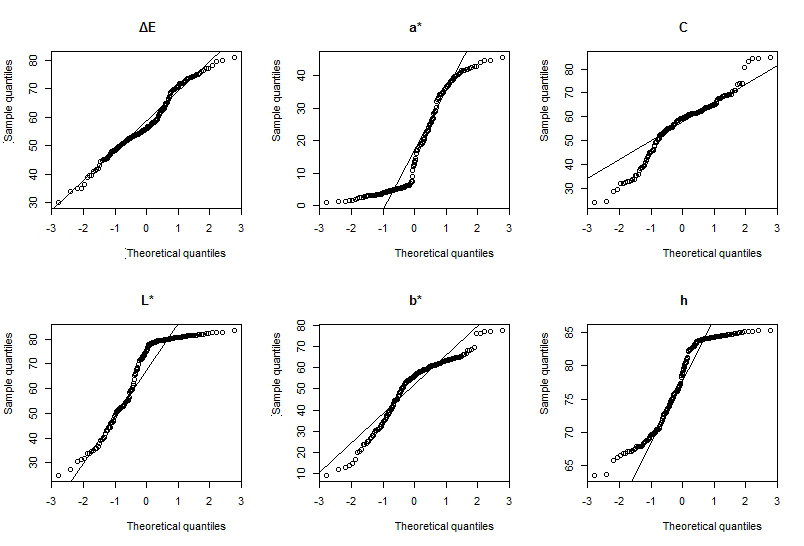

Supplement: S2 Fig — (TIF) [file pone.0216460.s002.tif]

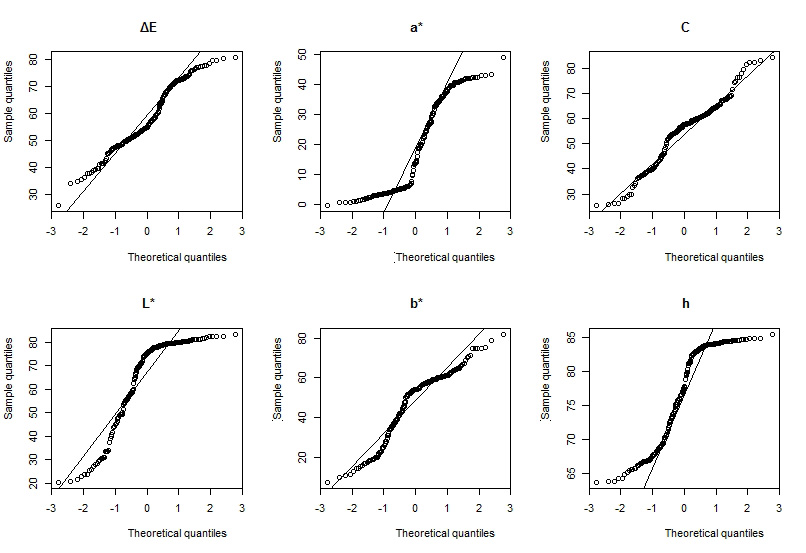

Supplement: S3 Fig — (TIF) [file pone.0216460.s003.tif]

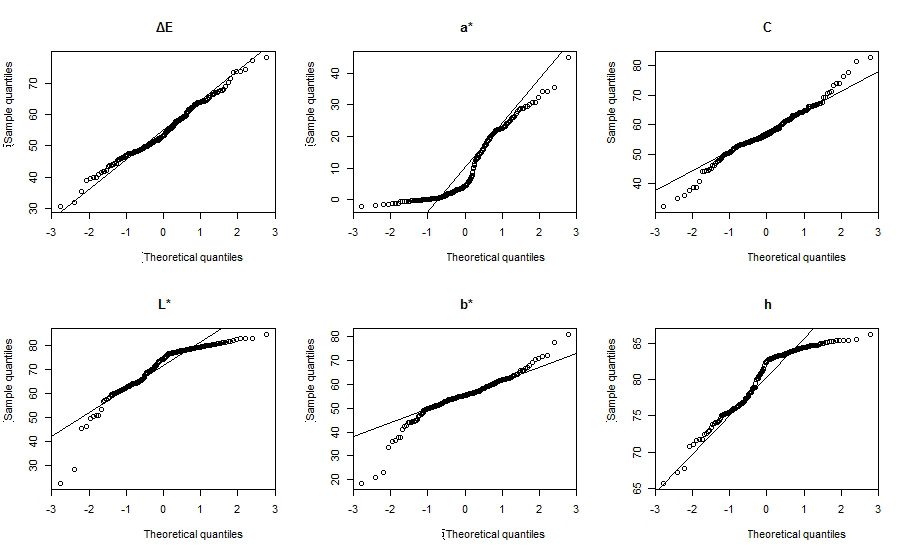

Supplement: S4 Fig — (TIF) [file pone.0216460.s004.tif]

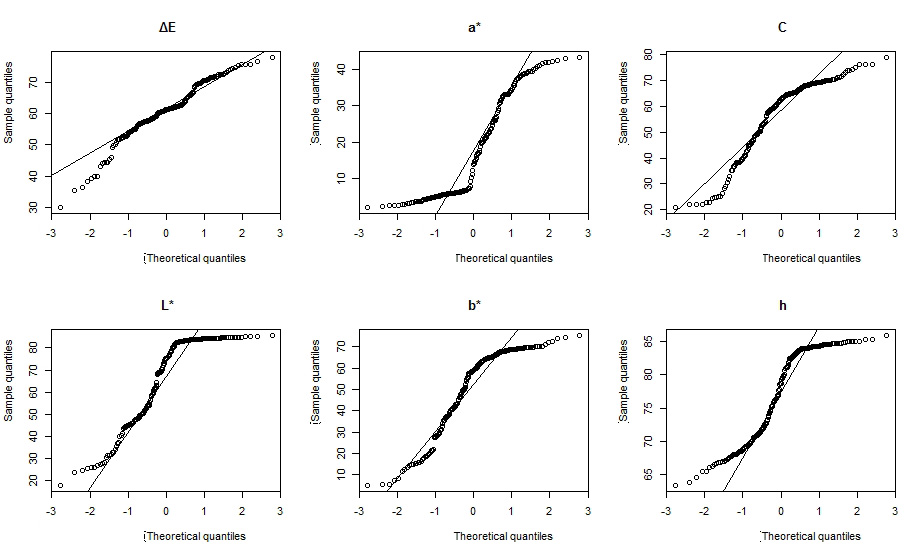

Supplement: S5 Fig — (TIF) [file pone.0216460.s005.tif]

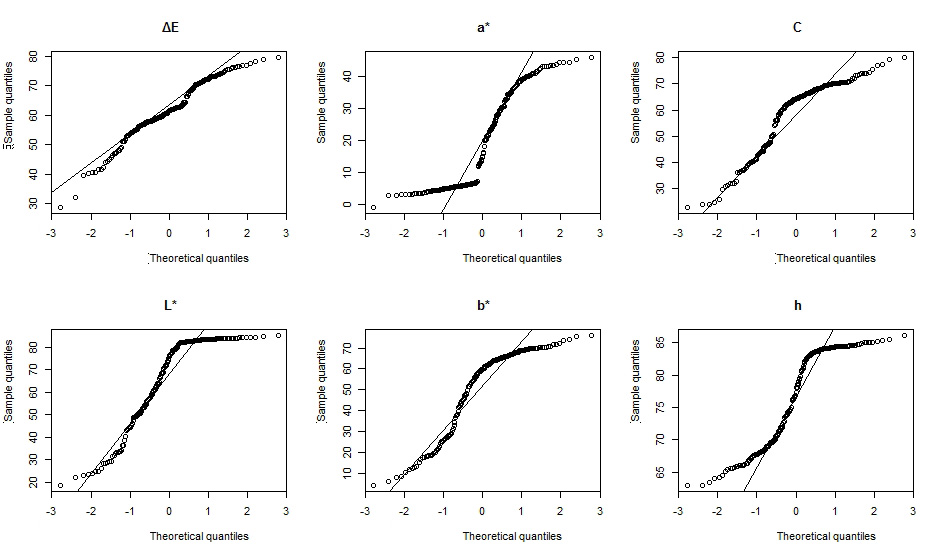

Supplement: S6 Fig — (TIF) [file pone.0216460.s006.tif]
