# Supplementary material for: The numerical classification and grading standards of daylily (Hemerocallis) flower color
Source: PLoS One. 2019 Jun 6;14(6):e0216460. doi: 10.1371/journal.pone.0216460 (PMC6553707; doi:10.1371/journal.pone.0216460)
Supplement: S1 Table — (DOCX) [file pone.0216460.s007.docx]

Table S1 The experimental Hemerocallis germplasms in this study

| No. | Name | Genetic Groups | No. | Name | Genetic Groups | No. | Name | Genetic Groups |
| --- | --- | --- | --- | --- | --- | --- | --- | --- |
| H0001 | ‘Suqian 1-H’ | 2 | H0075 | ‘Little Wine Cup’ | 2 | H0140 | ‘Frans Hals’ | 2 |
| H0002 | ‘Chazi Hua’ | 1 | H0076 | ‘Children’s Festival’ | 2 | H0141 | ‘Little Bumble Bee 2’ | 2 |
| H0003 | ‘Qiezi 1’ | 1 | H0077 | ‘Golden Doll 2’ | 2 | H0142 | ‘X-88’ | 3 |
| H0004 | ‘Pan Long Hua’ | 1 | H0078 | ‘Baltimore Oriole 2’ | 2 | H0143 | ‘X-56’ | 3 |
| H0005 | ‘Liu Yue Hua | 1 | H0079 | ‘Lusty Lealand’ | 2 | H0144 | ‘X-91’ | 3 |
| H0006 | ‘Datong’ | 1 | H0080 | ‘Ruby’ | 2 | H0145 | ‘Wenxi Xuancao’ | 2 |
| H0007 | *H. minor* Mill. | 1* | H0081 | ‘Bourbon King’ | 2 | H0146 | ‘Wenxi Huanghua’ | 1 |
| H0008 | *H. lilioasphodelus* L. | 1* | H0082 | ‘Sunset Chicago’ | 2 | H0147 | ‘Gaogan Chaiqiao Hua’ | 1 |
| H0009 | ‘Yesheng Huanghua 1’ | 1 | H0083 | ‘Dong Fang Bu Bai’ | 2 | H0148 | ‘Wuyue Hua’ | 1 |
| H0010 | ‘Qiaotou Huanghua ’ | 1 | H0084 | ‘Hong Bao’ | 2 | H0149 | ‘Laoqing Zaohua’ | 1 |
| H0013 | ‘Shijing Huanghua ’ | 1 | H0085 | ‘Xia Ri Jiu Hong’ | 2 | H0150 | ‘Paozhang Hua’ | 1 |
| H0014 | ‘Xiaoyu Huanghua ’ | 1 | H0086 | ‘Elegant Greeting’ | 2 | H0151 | ‘Qingzao Hua’ | 1 |
| H0015 | ‘Yesheng Xuancao 2’ | 1 | H0087 | ‘Purple Waters’ | 2 | H0152 | ‘Siyue Bai 2’ | 1 |
| H0016 | ‘Changzuizi Hua 1’ | 1 | H0088 | ‘Ma Lian’ | 2 | H0153 | ‘Xuebuxie Miao’ | 1 |
| H0017 | ‘Suqian 1-C’ | 2 | H0089 | ‘Orange’ | 2 | H0154 | ‘Gaogan Zhongqi Hua’ | 1 |
| H0018 | ‘Chongli Hua’ | 1 | H0090 | ‘Little Lassie’ | 2 | H0155 | ‘Daojian Hua 2’ | 1 |
| H0019 | ‘Shezhuang Huanghua ’ | 1 | H0091 | ‘Frans Hals 1’ | 2 | H0156 | ‘Changba Hua’ | 1 |
| H0020 | ‘Dali Huanghua ’ | 1 | H0093 | ‘American Ruby’ | 2 | H0157 | ‘Yuanqu Huanghua’ | 1 |
| H0021 | ‘Bai Hua’ | 1 | H0094 | ‘Austria Ruby’ | 2 | H0158 | *H. fulva* | 2* |
| H0022 | ‘Suqian 2-H’ | 2 | H0095 | ‘Dahua Xuancao’ | 2 | H0160 | ‘Huguan Huanghua’ | 1 |
| H0023 | ‘Suqian 3-H’ | 2 | H0096 | ‘Beijing 1’ | 3 | H0161 | ‘Zezhou Huanghua’ | 1 |
| H0024 | ‘Huaian 1’ | 2 | H0097 | ‘Beijing 2’ | 3 | H0162 | ‘Yingxian Huang hua’ | 1 |
| H0025 | ‘Huaian 2’ | 2 | H0098 | ‘Beijing 3’ | 2 | H0163 | ‘Anze Huanghua’ | 1 |
| H0026 | ‘Yesheng Xuancao 3’ | 2 | H0101 | ‘Little Bumble Bee 1’ | 2 | H0164 | ‘Gujiao Huanghua’ | 1 |
| H0027 | ‘Xian Huanghua 1’ | 1 | H0102 | ‘Beijing 7’ | 3 | H0166 | ‘Gaoping Huanghua’ | 1 |
| H0028 | *H. minor* Mill. | 1* | H0103 | ‘Beijing 8’ | 3 | H0167 | ‘Gansu Huanghua’ | 1 |
| H0029 | ‘Malin Huanghua ’ | 1 | H0104 | ‘Beijing 9’ | 3 | H0168 | ‘Xuanhua Huanghua’ | 1 |
| H0030 | ‘Huo Huanghua ’ | 1 | H0105 | ‘Dandong Xuancao’ | 2 | H0169 | ‘Guangling Huanghua 2’ | 1 |
| H0031 | ‘Gaoting Huanghua ’ | 1 | H0106 | ‘Chang Zuizi Hua 2’ | 1 | H0170 | ‘Datong Huanghua 3’ | 1 |
| H0032 | ‘Green Mystique’ | 2 | H0107 | ‘Xianju Huanghua’ | 1 | H0171 | ‘Guangling Huanghua 3’ | 1 |
| H0033 | ‘ Chicago Apache’ | 2 | H0108 | ‘Chongli Hua 2’ | 1 | H0172 | ‘Lanfeng Huanghua’ | 1 |
| H0034 | ‘Veins and Truth’ | 2 | H0109 | ‘Gulao Wuming Hua’ | 1 | H0173 | ‘Suizhou Huanghua’ | 1 |
| H0035 | ‘Double Cutie’ | 2 | H0110 | ‘Zhongqi Hua’ | 1 | H0174 | ‘Bishan Huang Hua’ | 1 |
| H0036 | ‘Moonlit Masquerade’ | 2 | H0111 | ‘Panlong Hua 2’ | 1 | H0176 | ‘Chifeng Huanghua’ | 1 |
| H0037 | ‘Baltimore Oriole 1’ | 2 | H0112 | ‘Hei Zuizi Hua’ | 1 | H0178 | ‘Yuanqu Huanghua’ | 3 |
| H0039 | ‘Dongzhuang Huanghua ’ | 1 | H0113 | ‘Zao Huanghua’ | 1 | H0179 | ‘Yanchi Huanghua’ | 1 |
| H0040 | *H. aurantiaca* Baker. | 2* | H0114 | ‘Bai Hua 2’ | 1 | H0181 | ‘Hukou Huanghua’ | 1 |
| H0042 | *H. thunbergerii* | 1* | H0115 | ‘Chashan Tiaozi Hua’ | 1 | H0182 | ‘Wennan 1’ | 1 |
| H0044 | *H. hakuunensis* Nakai. | 2* | H0116 | ‘Bayue Hua’ | 1 | H0183 | ‘Wennan 2’ | 1 |
| H0045 | *H. multiflora* Stout. | 2* | H0117 | ‘Tianguang Hua’ | 1 | H0185 | ‘Little Red Baron’ | 2 |
| H0046 | *H. altissima* Stout. | 2* | H0118 | ‘Longyou Honghua’ | 2 | H0186 | ‘Hunse’ | 2 |
| H0047 | *H. citrina* Baroni. | 1* | H0119 | ‘Yesheng Xuancao 4’ | 2 | H0187 | ‘Canadian Border Patrol’ | 2 |
| H0052 | *H. fulva* var. *kwanso* var. *reasata* | 2* | H0120 | ‘Huaiyang Hua’ | 1 | H0188 | ‘Daring Dilemma’ | 2 |
| H0054 | ‘Autumn Red’ | 2 | H0121 | ‘Dingzhuang Dacai’ | 1 | H0189 | ‘James Marsh’ | 2 |
| H0056 | ‘Rocket City’ | 2 | H0122 | ‘Siyue Bai’ | 1 | H0190 | ‘Chicago Apache’ | 2 |
| H0057 | ‘Purple Gems’ | 2 | H0123 | ‘Quxian Huanghua’ | 1 | H0191 | ‘Li Chuan Huang Hua’ | 1 |
| H0058 | ‘Pink Damask’ | 2 | H0124 | ‘Tai Dong 6 ’ | 1 | H0192 | ‘Xin Yi Huang Hua’ | 1 |
| H0059 | ‘Da Huang Hua Za’ | 1 | H0125 | ‘Malin Huanghua 2’ | 3 | H0193 | ‘Datong Xin Huanghua’ | 1 |
| H0060 | ‘Blazing sun’ | 2 | H0126 | ‘Shayuan Jinzhen’ | 1 | H0194 | ‘17T’ | 3 |
| H0061 | ‘Crimson Pirate’ | 2 | H0127 | ‘Siyue Honghua’ | 2 | H0195 | ‘Weizhi Huanghua’ | 3 |
| H0062 | ‘Blue Sheen 1’ | 2 | H0128 | ‘Mengzi Hua’ | 1 | H0201 | ‘Lace Doily’ | 2 |
| H0063 | ‘Xiao Hong 74’ | 2 | H0129 | ‘Wuping Zao’ | 1 | H0202 | ‘Anna’ | 2 |
| H0064 | ‘Bonanza’ | 2 | H0130 | ‘Jingzhou Hua’ | 1 | H0203 | ‘Pink Paradise’ | 2 |
| H0065 | ‘Lullaby Baby’ | 2 | H0132 | ‘Datong Huanghua 2’ | 1 | H0204 | ‘Lavender Tutu’ | 2 |
| H0066 | ‘Canadian Border Patrol’ | 2 | H0133 | ‘Chaiqiao Hua’ | 1 | H0205 | ‘Always Afternoon’ | 2 |
| H0067 | ‘Red Cloud’ | 2 | H0134 | ‘Daojian Hua’ | 1 | H0206 | ‘Entrapment’ | 2 |
| H0069 | ‘Toxic Qiu.H’ | 2 | H0135 | ‘Xian Huanghua 2’ | 1 | H0207 | ‘Forty second street’ | 2 |
| H0070 | ‘Betty wods’ | 2 | H0136 | ‘Da Wuzui’ | 1 | H0208 | ‘Mildred Mitchell’ | 2 |
| H0071 | ‘Fen Yuan’ | 2 | H0137 | ‘Xiyezi Hua’ | 1 | H0209 | ‘French Lingerie’ | 2 |
| H0072 | ‘H400’ | 2 | H0138 | ‘Qiezi Hua 2’ | 1 | H0210 | ‘Lavender Deal’ | 2 |
| H0073 | ‘Little Grapette’ | 2 | H0139 | ‘Golden Doll 3’ | 2 |  |  |  |
| H0074 | ‘Z-300’ | 2 |  |  |  |  |  |  |

Note: 1 denotes edible day lily, 2 denotes horticultural cultivars, 3 denotes breeding lines; * denotes wild species
